# Supplementary material for: Single-cell mRNA-regulation analysis reveals cell type-specific mechanisms of type 2 diabetes
Source: Nat Commun. 2025 Oct 27;16:9475. doi: 10.1038/s41467-025-65060-z (PMC12559761; doi:10.1038/s41467-025-65060-z)
Supplement: Supplementary file 1 — Supplementary Information [file 41467_2025_65060_MOESM1_ESM.pdf]

SUPPLEMENTARY INFORMATION

Compiled for:

Single-cell mRNA-regulation analysis reveals cell type-specific mechanisms of type 2 diabetes

Martínez-López J.A.[1, 2#], Lindqvist A.[3#], Lopez-Pascual A.[3],[#] Harder A.[1,4], Chen P.[5], Ngara M.[3], Shcherbina L.[3], Siffo S. [3], Cowan E. [6], Baira S.M. [7], Kryvokhyzha D. [6], Karagiannopoulos A. [6], Chriett S.[3], Skene N.G.[1], Prasad R.B.[6], Lancien M.[8], Johnson P.F.[9], Eliasson P.[10], Eliasson L. [6], Louvet C.[8], Spégel P. [7], Muñoz-Manchado A.B.[1,11], Sandberg R.[12], Hjerling-Leffler J.[1,§*], Wierup N.[3, 13§*]

[1]Laboratory of Molecular Neurobiology, Department of Medical Biochemistry and Biophysics, Karolinska Institutet, Stockholm, Sweden

[2] Department of Engineering, Universidad Loyola, Seville, Spain

[3]Lund University Diabetes Centre, Department of Experimental Medical Science, Lund University, Malmö, Sweden

[4] Department of Medical Epidemiology and Biostatistics, Karolinska Institutet, Stockholm, Sweden

[5]Division of Clinical Chemistry, Department of Laboratory Medicine, Karolinska Institutet, Stockholm, Sweden

[6]Lund University Diabetes Centre, Department of Clinical Sciences in Malmö, Lund University, Malmö, Sweden

[7] Department of Chemistry, Centre for Analysis and Synthesis, Lund University, Lund, Sweden

[8] INSERM UMR 1064, Center for Transplantation and Immunology, Université de Nantes, Nantes, France

[9] Mouse Cancer Genetics Program, Center for Cancer Research, NCI, Frederick, MD, USA

[10]Bioscience Cardiovascular, Research and Early Development, Cardiovascular, Renal and Metabolism (CVRM), Biopharmaceuticals R&D, AstraZeneca, Gothenburg, Sweden.

[11] Departamento de Anatomía Patológica, Biología Celular, Histología, Historia de la Ciencia, Medicina Legal y Forense y Toxicología. Instituto de Investigación e Innovación Biomédica de Cádiz (INiBICA). Universidad de Cádiz, Spain

[12] Department of Cell and Molecular Biology, Karolinska Institutet, Stockholm, Sweden

[13] Scania University Hospital, Clinical Research Centre, Malmö, Sweden

§ Correspondence to: nils.wierup@med.lu.se and jens.hjerling-leffler@ki.se

* These authors jointly supervised this work

# These authors contributed equally

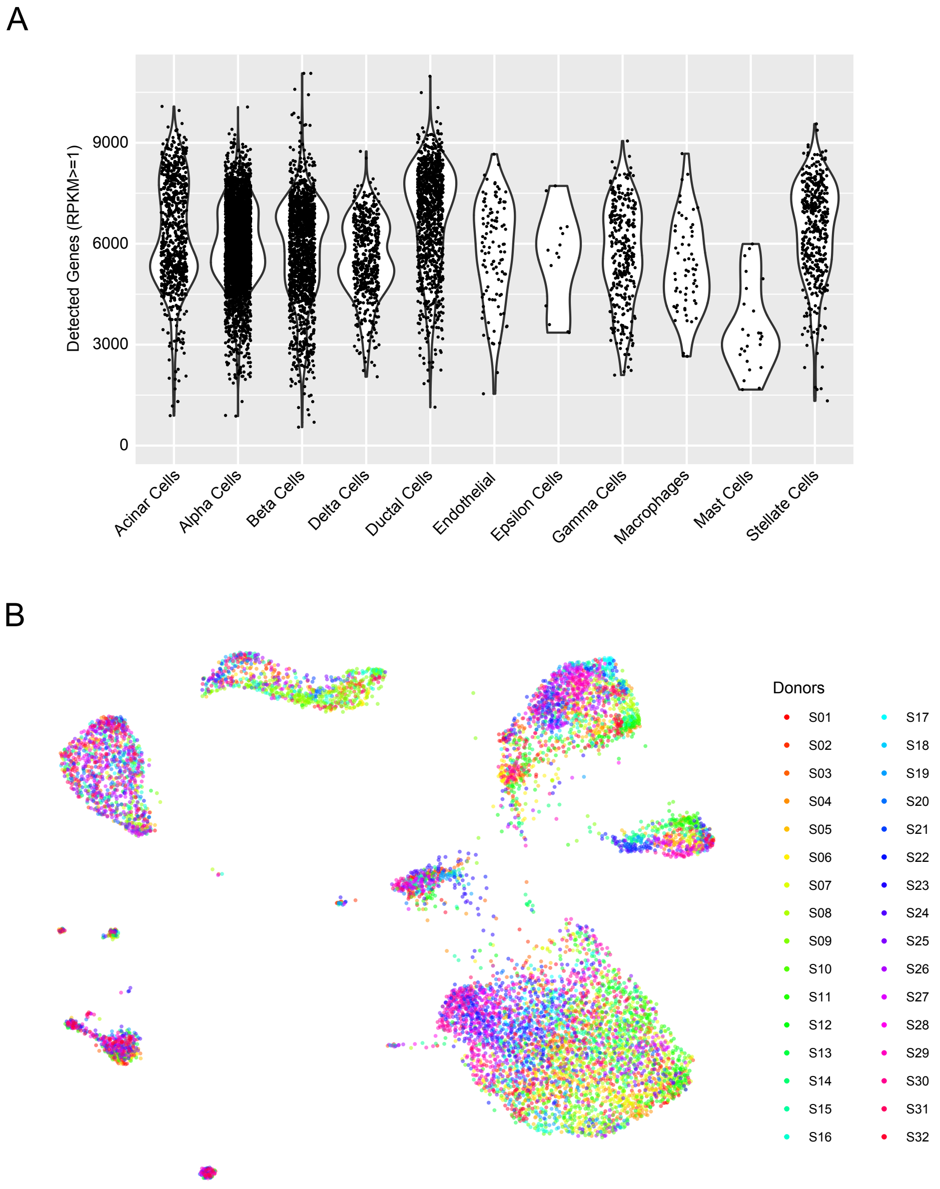

Figure S1. Number of genes detected and individual donors indicated in cell type clustering.

A Number of detected genes per cell type. **B** UMAP of 8511 cells with donors indicated.

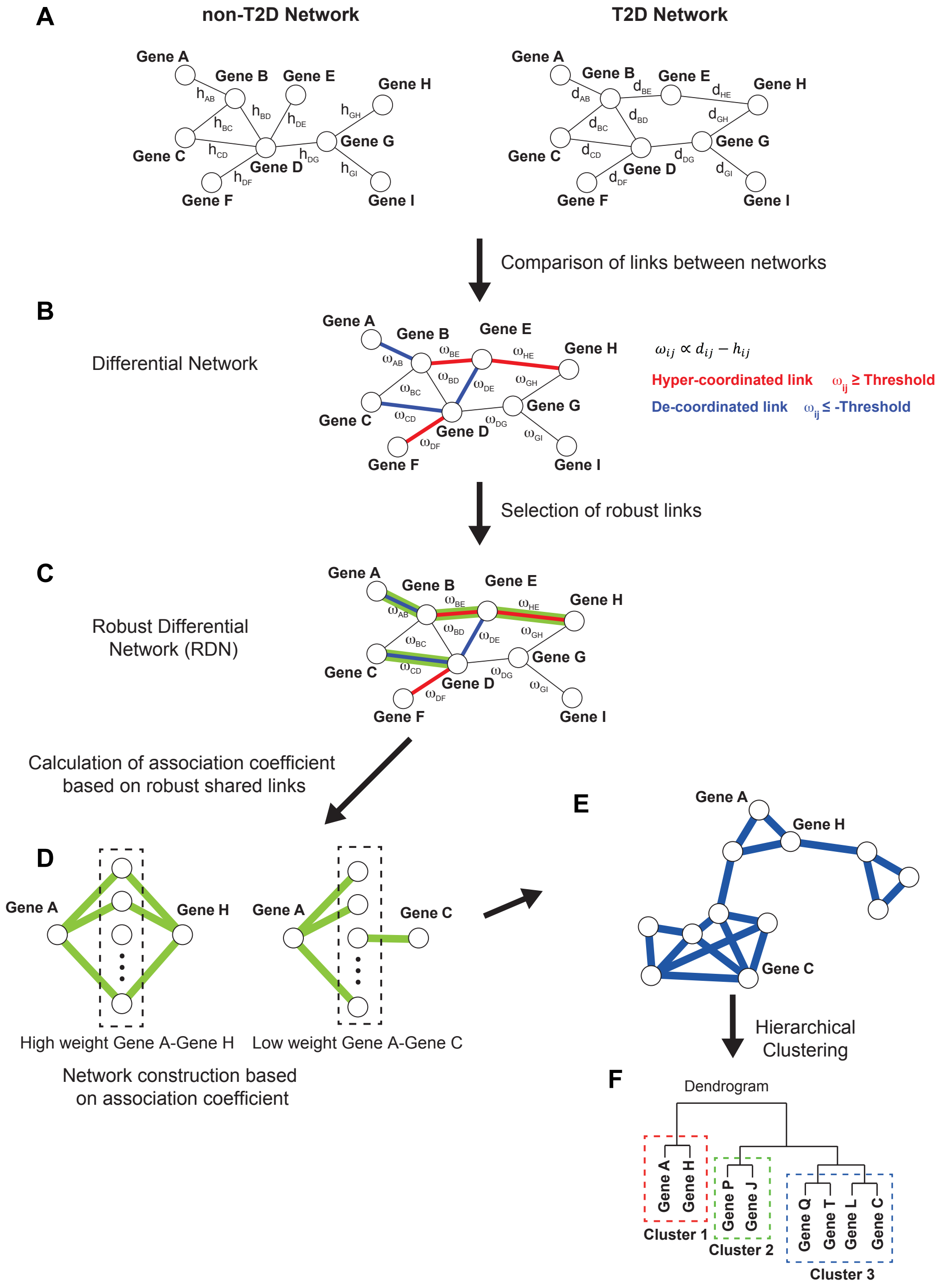
A
non-T2D Network
T2D Network
Gene A
Gene B
Gene C
Gene D
Gene E
Gene F
Gene G
Gene H
Gene I
Comparison of links between networks
B
Differential Network
ωij ∝ dij − hij
Hyper-coordinated link ωij ≥ Threshold
De-coordinated link ωij ≤ -Threshold
Selection of robust links
C
Robust Differential Network (RDN)
Calculation of association coefficient based on robust shared links
D
High weight Gene A-Gene H
Low weight Gene A-Gene C
Network construction based on association coefficient
E
Hierarchical Clustering
F
Dendrogram
Gene P
Gene J
Gene Q
Gene T
Gene L
Cluster 1
Cluster 2
Cluster 3

Figure S2. Overview of differential gene coordination network analysis (dGCNA).

A Creation of two matrices (one for each state; T2D/non-T2D) containing all gene-gene correlations determined as the residuals of a mixed model controlling for donor identity. **B** Comparison of the two matrices creating a delta-matrix based on change in correlation. **C** Creation of a robust coordination network by selection of significant changes, based on bootstrapping analysis with random connections. **D** Calculation of association coefficients based on the robust differential network (RDN). **E** Topological analysis and network construction. **F** Hierarchical clustering of networks.

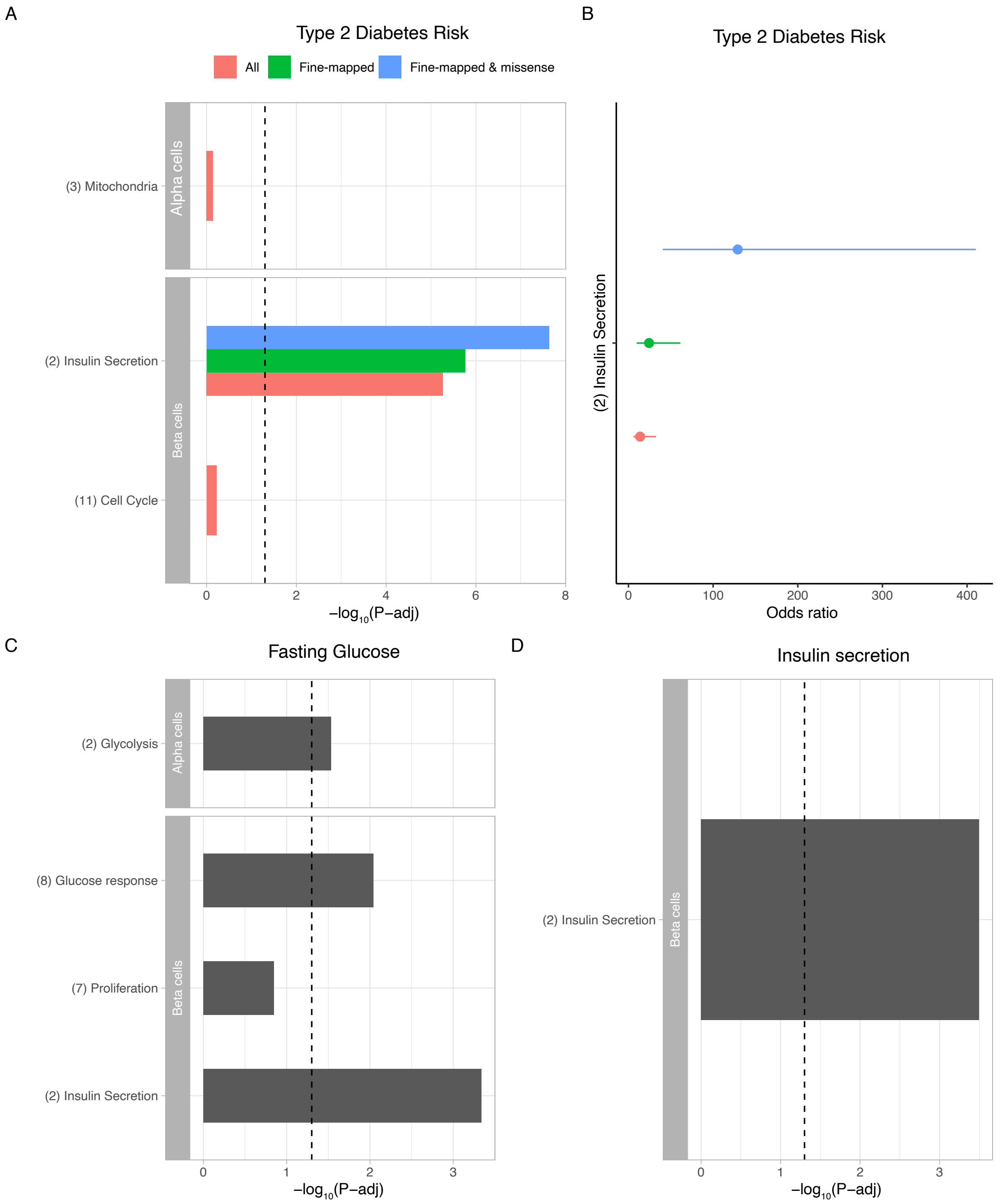

Figure S3. Gene-set enrichment analysis (GSEA) for GWAS genes in dGCNA gene modules.

Gene-set enrichment analysis (GSEA) with Fisher's exact test. Y-axes marks dGCNA gene modules. **A-B** Analysis of three gene-sets of type 2 diabetes risk GWAS as indicated in Results (All genetically implicated genes, fine-mapped genes, and fine-mapped genes with a missense mutation). **A** FDR corrected p-value for alpha- and beta cell modules with significant enrichment of GWAS genes. **B** Estimated odds ratio for each set of GWAS genes with the Insulin Secretion module in **A**. **C-D** FDR corrected p-value from GSEA with dGCNA modules with GWAS genes for fasting glucose and insulin secretion respectively.

A

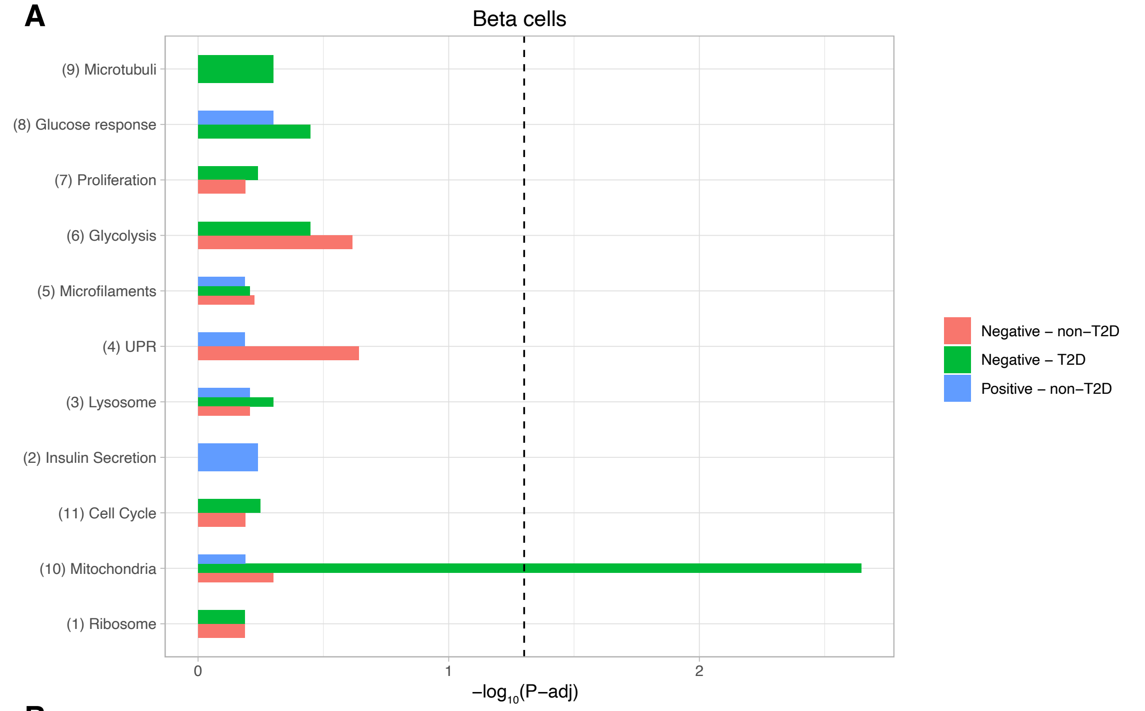

B

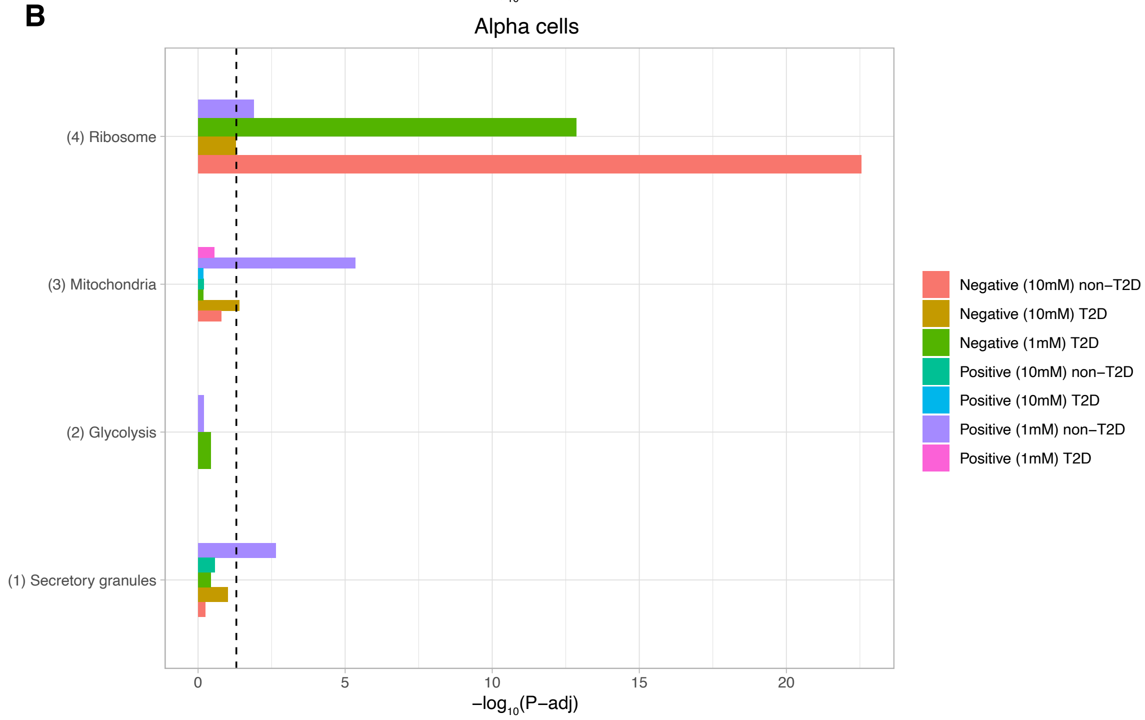

Figure S4. Gene-set enrichment analysis (GSEA) for exocytosis-related genes in beta- and alpha cell dGCNA gene modules

A GSEA of genes related to exocytosis in beta cells. **B** GSEA of genes related to exocytosis in alpha cells. The y-axes display the dGCNA modules and the X-axes display the -$\log_{10}$ FDR adjusted p-value. The minimum overlap size was set to >= 2. Exocytosis-related genes in alpha- and beta cells were split by the sign of the correlation coefficient (negative or positive), as well as by donor status (T2D vs non-T2D), resulting in four gene-sets tested in beta cells. In B gene-sets were split additionally by glucose concentration (1mM vs 10mM), resulting in eight gene-sets tested in alpha cells.

A

B

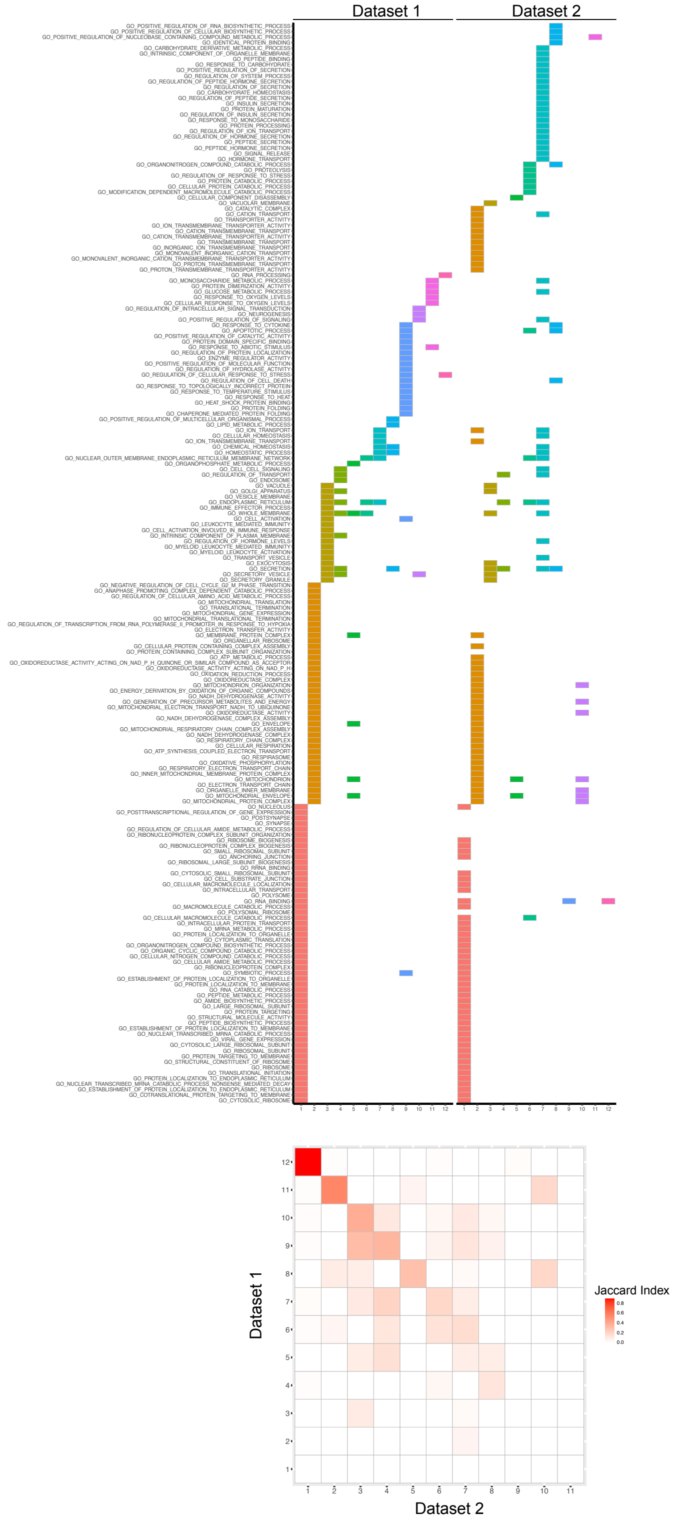

Figure S5. Analysis of overlap of GO-terms for dataset 1 and dataset 2

A GSEA for GO-terms and genes in each module detected by dGCNA, in dataset 1 and dataset 2 separately. Left panel shows all GO-terms detected across all modules in dataset 1. Right panel shows all GO-terms detected for modules in dataset 2. The Y-axis marks each GO-term, the X-axis marks the module, the panel shows which dataset the module was found in, and a colored square means that GSEA detected an FDR-adjusted significant overlap between the GO-term and the dGCNA module.

B Heatmap of overlap in terms of significant GO-terms, between the different modules detected in dataset 1 and dataset 2. The squares are colored by the Jaccard Index (intersection divided by union) of GO-terms detected for each pair of modules in dataset 1 and dataset 2.

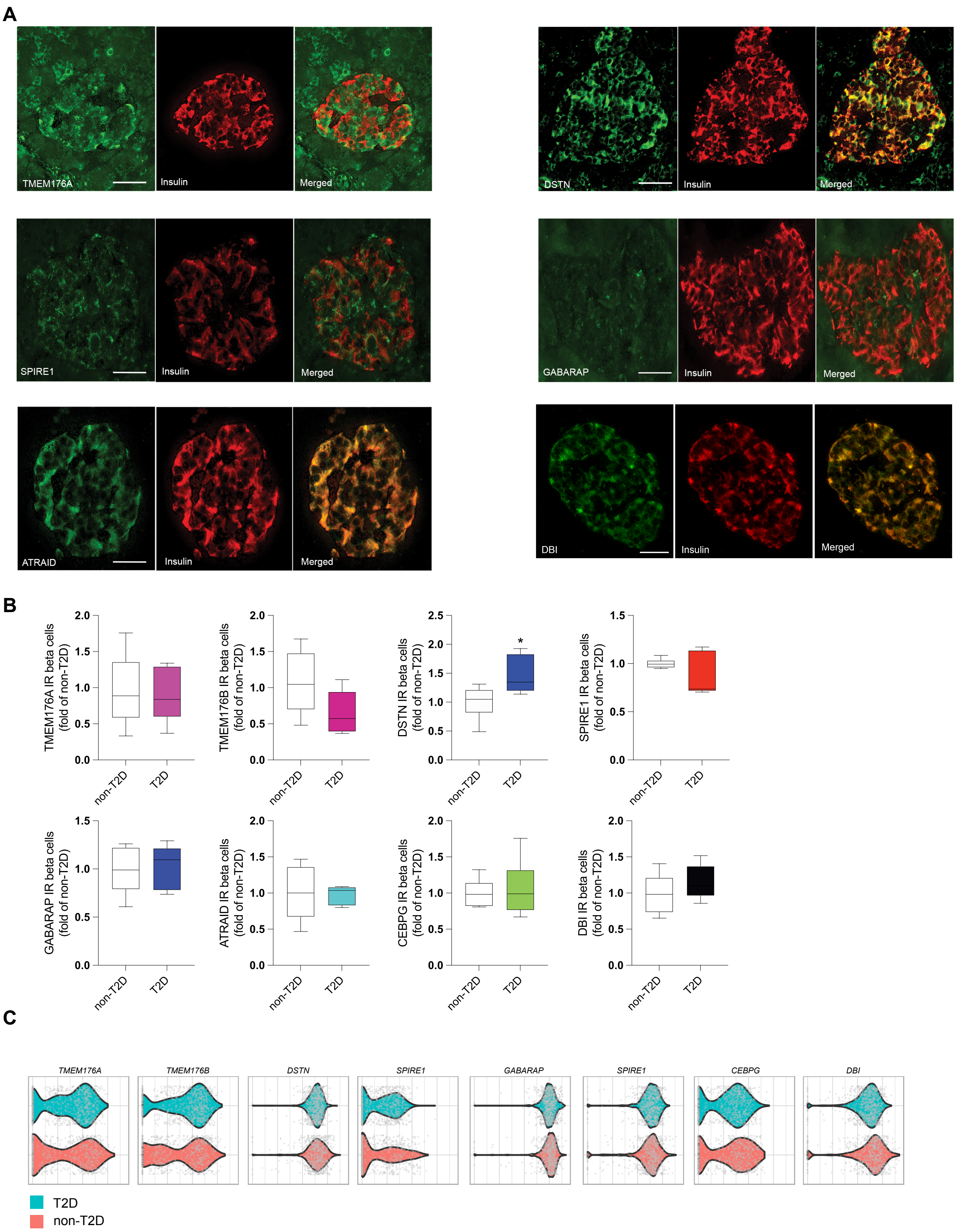

Figure S6. Immunostaining and quantification of beta cell immunoreactivity in non-T2D vs. T2D donors, and beta cell mRNA expression of genes selected for functional analysis.

A Double immunostaining for target gene protein products and insulin in human pancreatic sections shows expression of TMEM176A (top left panels), DSTN (top right panels), SPIRE1 (middle left panels), GABARAP (middle right panels), and ATRAID (bottom left panels) and DBI (bottom right panels) in human beta cells. Representative images. 50 islets from 5 donors were analyzed. Scale bars=50 μm. **B** Morphometric quantification of beta cell protein

expression levels (immunoreactive beta cells) in T2D (n=5 for all except CEBPG which had n=6) vs. non-T2D (n=6) donors. Data is presented as box plots. Hinges of the box represent 25th and 75th percentile and the line in the box is the median. Whiskers are min and max values. * $p<0.05$. Two-tailed Student's t-test was used. **C** Gene expression values in $\log_{10}$(RPKM +1) from Beta cells in the merged dataset (n=32 donors). Eight genes selected for functional analysis are displayed; *ATRAID, CEBPG, DBI, DSTN, GABARAP, SPIRE1, TMEM176A, TMEM176B*. Gene expression and distributions are shown separated by donor status. None of the eight genes were DEGs in the differential gene expression analysis (P-adj > 0.05). Source data are provided as a Source Data file.

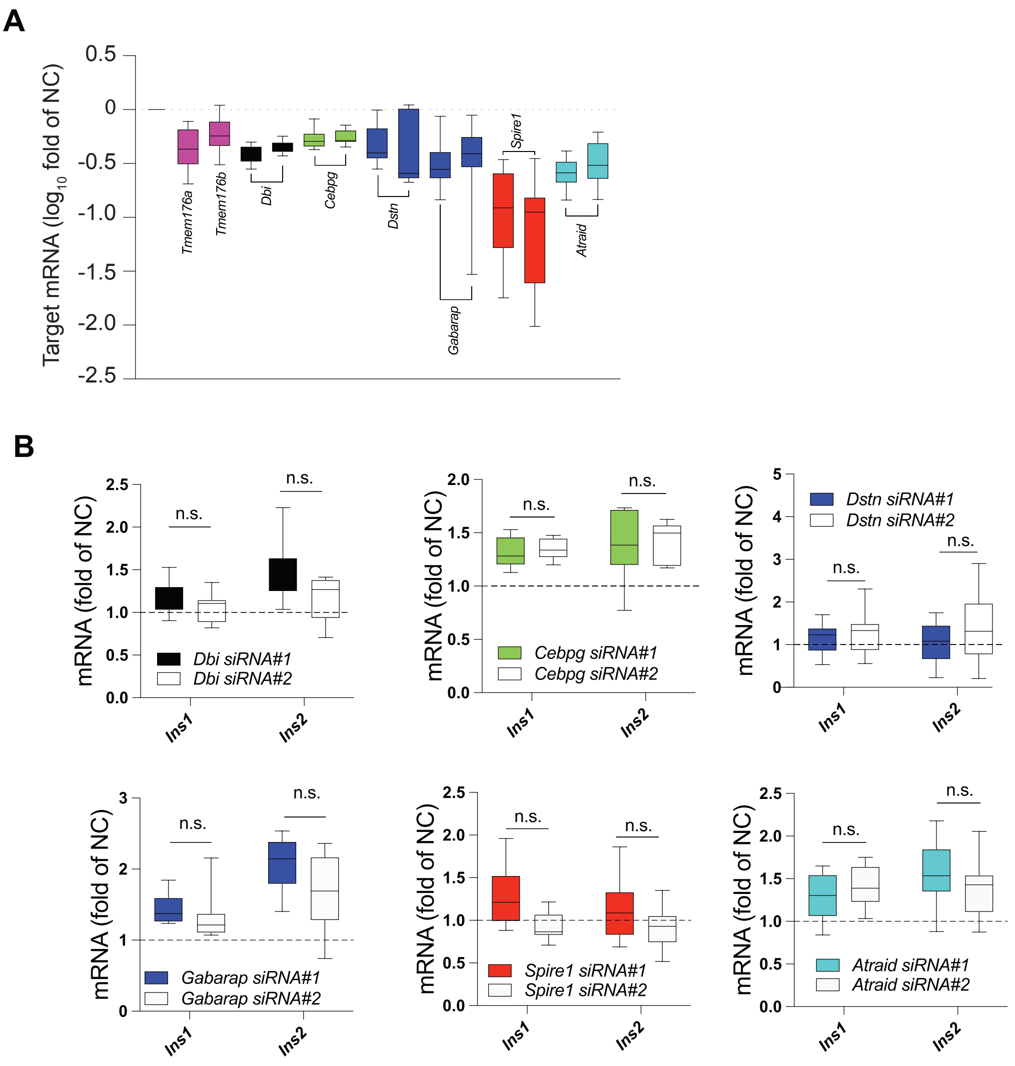

Figure S7. Knockdown efficiency and effect of individual pairs of siRNAs on insulin mRNA expression in INS-1 832/13 cells.

A Knockdown efficiency of each pair of siRNAs used. All pairs of siRNA significantly ($p<0.05$) reduced the expression of the gene of interest compared with scrambled control siRNA (dashed line), n=6 per condition. **B** Effect of each pair of siRNAs on *Ins1* and *Ins2* mRNA expression, n=6 per condition. Data is presented as box plots. Hinges of the box represent 25th and 75th percentile and the line in the box is the median. Whiskers are min and max values. Two-tailed Student's t-test was used. Source data are provided as a Source Data file.

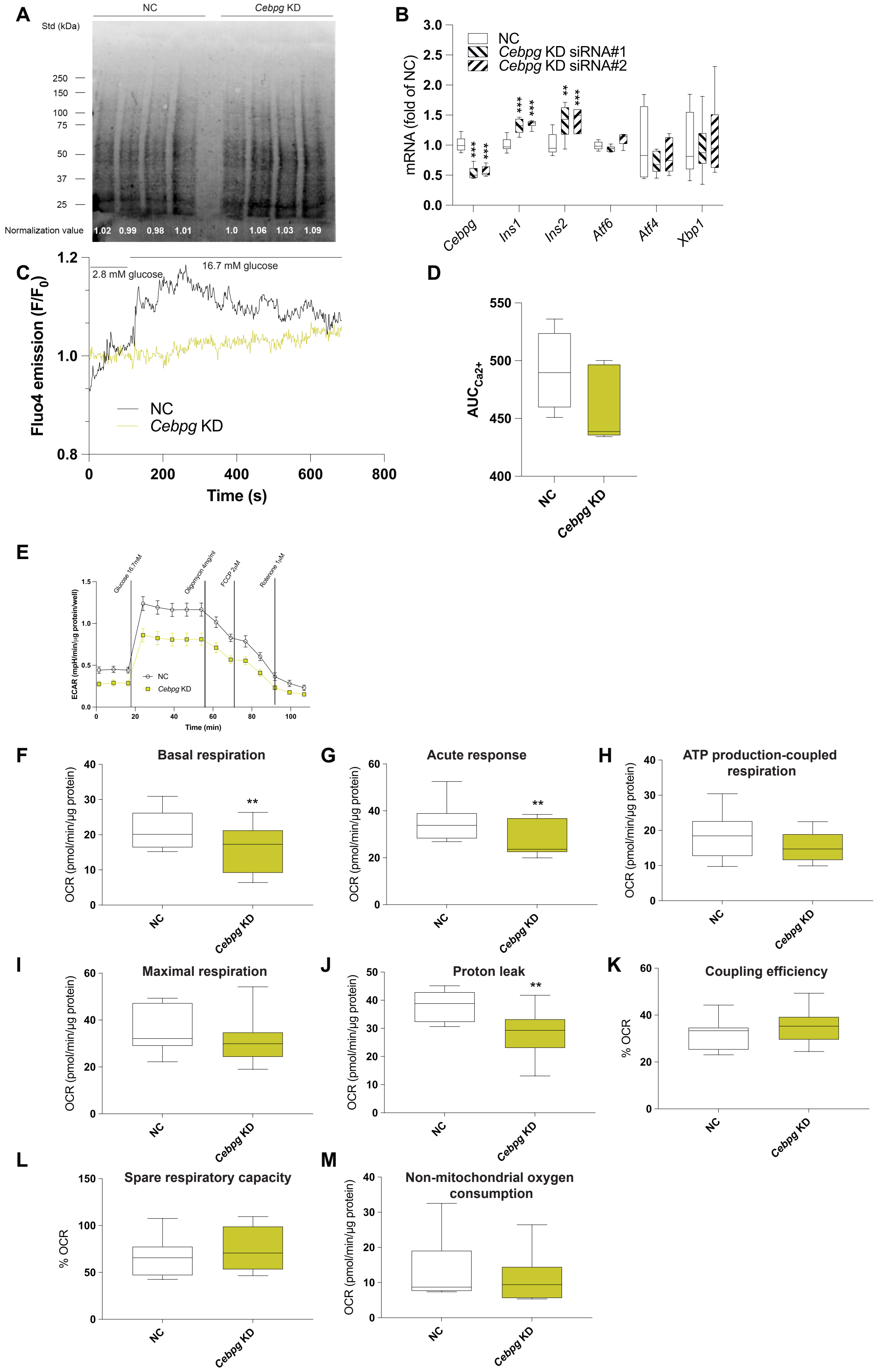
A
NC
Cebpg KD
Std (kDa)
250
150
100
75
50
37
25
Normalization value
1.02 0.99 0.98 1.01
1.0 1.06 1.03 1.09
B
NC
Cebpg KD siRNA#1
Cebpg KD siRNA#2
mRNA (fold of NC)
Cebpg
Ins1
Ins2
Atf6
Atf4
Xbp1
C
2.8 mM glucose
16.7 mM glucose
Fluo4 emission (F/F0)
NC
Cebpg KD
Time (s)
D
AUCCa2+
NC
Cebpg KD
E
Glucose 16.7mM
Oligomycin 4mg/ml
FCCP 2uM
Rotenone 1uM
ECAR (mpH/min/μg protein/well)
NC
Cebpg KD
Time (min)
F
Basal respiration
OCR (pmol/min/μg protein)
G
Acute response
H
ATP production-coupled respiration
I
Maximal respiration
J
Proton leak
K
Coupling efficiency
% OCR
L
Spare respiratory capacity
M
Non-mitochondrial oxygen consumption

Figure S8. Extended *Cebpg* data in INS-1 832/13 cells.

A Loading control for Fig 5G. Normalization values for protein loading indicated. **B** Effect of *Cebpg* KD on mRNA expression of insulin (*Ins1* and *Ins2*), and unfolded protein response regulators in the absence of thapsigargin, n=6 per condition. **C** *Cebpg* knockdown does not affect glucose-induced Ca^{2+} levels ; AUC in **(D)**, n=5 per condition. **E-M** Seahorse experiments showing *Cebpg* knockdown to reduce basal respiration (**F**), acute response (**G**), and proton leak (**J**). *Cebpg* knockdown had no effect on ATP production-coupled respiration (**H**), maximal respiration (**I**), coupling efficiency (**K**), spare respiratory capacity (**L**), or non-mitochondrial oxygen consumption (**M**). E-M n=8 per condition. Data in B, D, F-M is presented as box plots. Hinges of the box represent 25th and 75th percentile and the line in the box is the median. Whiskers are min and max values. ** p<0.01, *** p<0.001. Two-tailed Student's t-test was used. Source data are provided as a Source Data file.

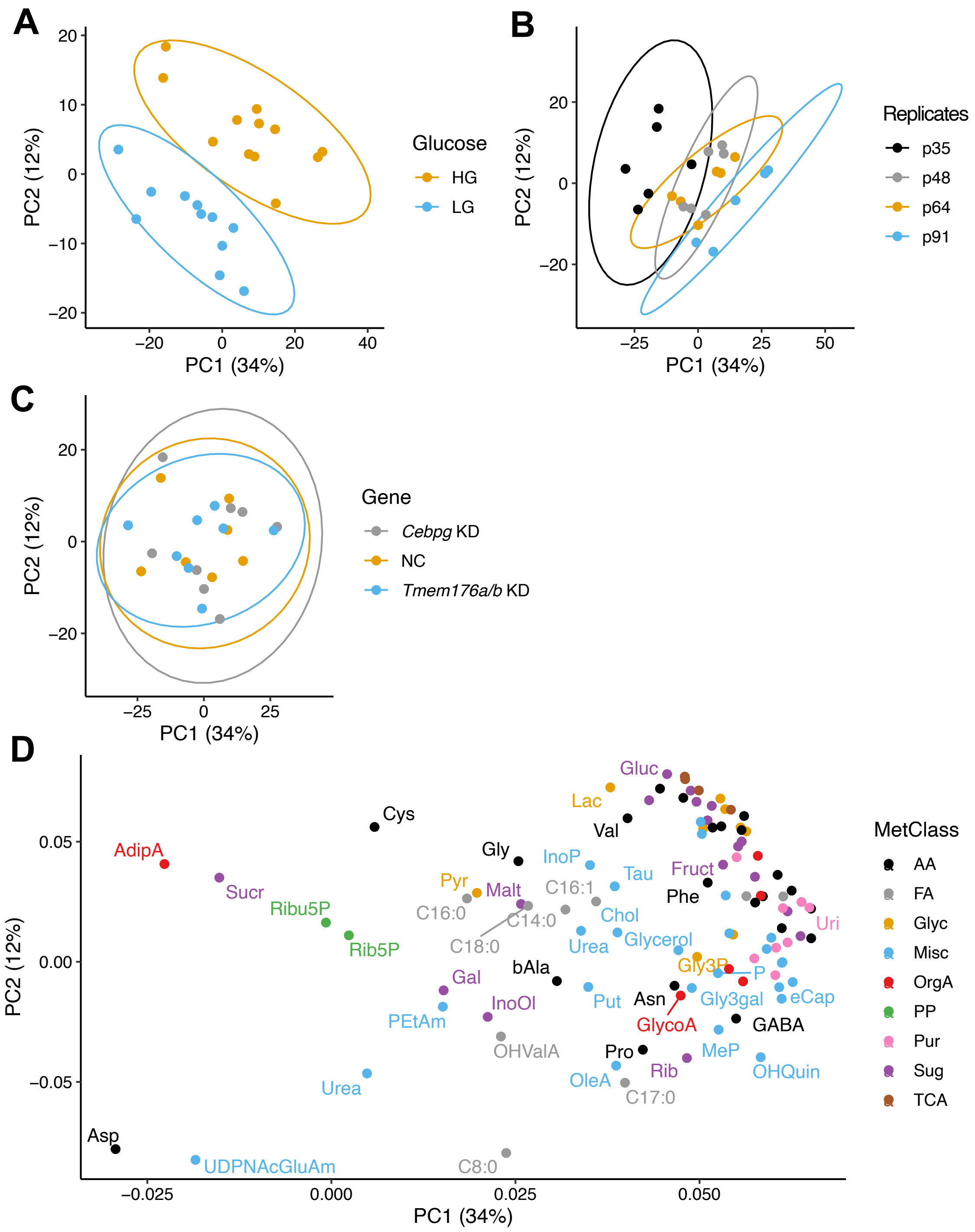

Figure S9. Metabolite profiling in INS-1 832/13 cells.

A-C Score-scatter plots showing glucose level (2.8 mM, LG; 16.7 mM, HG) **(A)** and cell line passage replicates **(B)**, but not knockdown of *Cebpg* or *Tmem176a/b* **(C)** to impact on the INS-1 832/13 metabolome. The first two principal components (PCs) are shown, with explained variance expressed within parantheses. **D** Expected glucose-induced metabolite alterations, including up-regulation of the metabolite classes (MetClass) glycolysis (Glyc), tricarboxylic acid (TCA) cycle as well as glutamate, and down-regulation of aspartate, were

observed. Only identified metabolites are shown; not all metabolites are named to enhance the clarity of the plot. In total 199 metabolite features were significantly impacted by glucose, 327 by cell population and none by *Cebpg* or *Tmem176a/b* knockdown (FDR<0.05). No significant interactions were found for *Cebpg* or *Tmem176a/b* knockdown with glucose level (FDR>0.05). n=4 per condition. Source data are provided as a Source Data file.

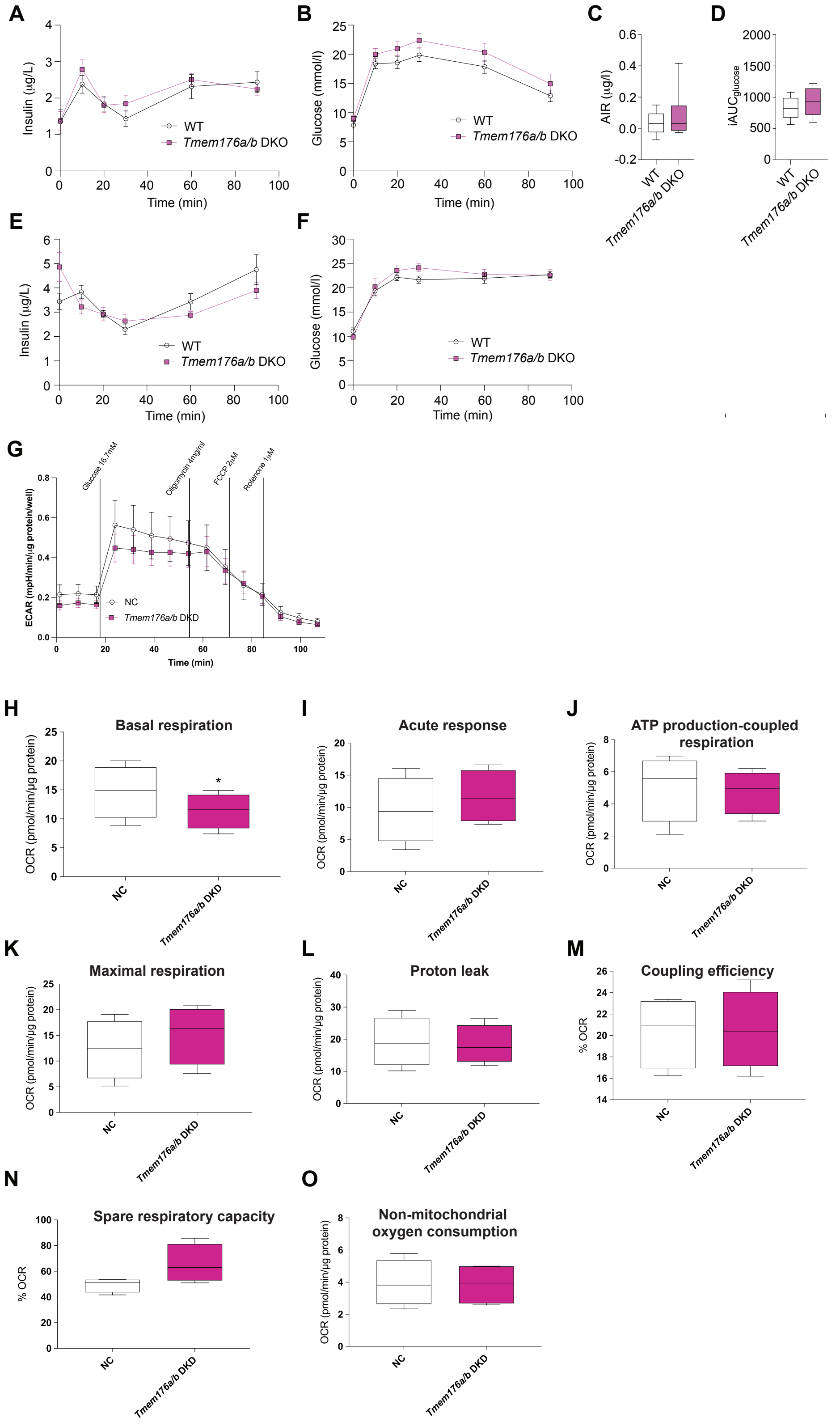

A
B
C
D
E
F
G
H
I
J
K
L
M
N
O
Insulin (µg/L)
Glucose (mmol/l)
Time (min)
WT
Tmem176a/b DKO
AIR (µg/l)
iAUCglucose
Glucose 16.7mM
Oligomycin 4mg/ml
FCCP 2µM
Rotenone 1µM
ECAR (mpH/min/µg protein/well)
NC
Tmem176a/b DKD
Basal respiration
Acute response
ATP production-coupled respiration
Maximal respiration
Proton leak
Coupling efficiency
Spare respiratory capacity
Non-mitochondrial oxygen consumption
OCR (pmol/min/µg protein)
% OCR

Figure S10. Extended *Tmem176a/b* data.

A-D Intraperitoneal glucose tolerance test (IpGTT) in *Tmem176a/b* DKO mice fed a normal diet. No difference detected with respect to insulin **(A)** and glucose **(B)** levels, acute insulin response (AIR) **(C)** or iAUC for glucose **(D)**. **E-F** IpGTT in *Tmem176a/b* DKO mice fed a high-fat diet. *Tmem176a/b* DKO had a negative insulin response (**E**), (AIR in **Fig. 6D**) and higher postprandial glucose levels (**F**), (iAUC in **Fig. 6E**). A-E: n=10 per group. **G-O** Seahorse experiments in INS-1 832/13 cells. *Tmem176a/b* knockdown reduces basal respiration (**H**). *Tmem176a/b* knockdown has no effect on acute response (**I**), ATP production-coupled respiration (**J**), maximal respiration (**K**), proton leak (**L**), coupling efficiency (**M**), spare respiratory capacity (**N**), or non-mitochondrial oxygen consumption (**O**). n=4 per condition. Data in C, D, H-O is presented as box plots. Hinges of the box represent 25th and 75th percentile and the line in the box is the median. Whiskers are min and max values. * $p<0.05$. Two-tailed Student's t-test was used. Source data are provided as a Source Data file.

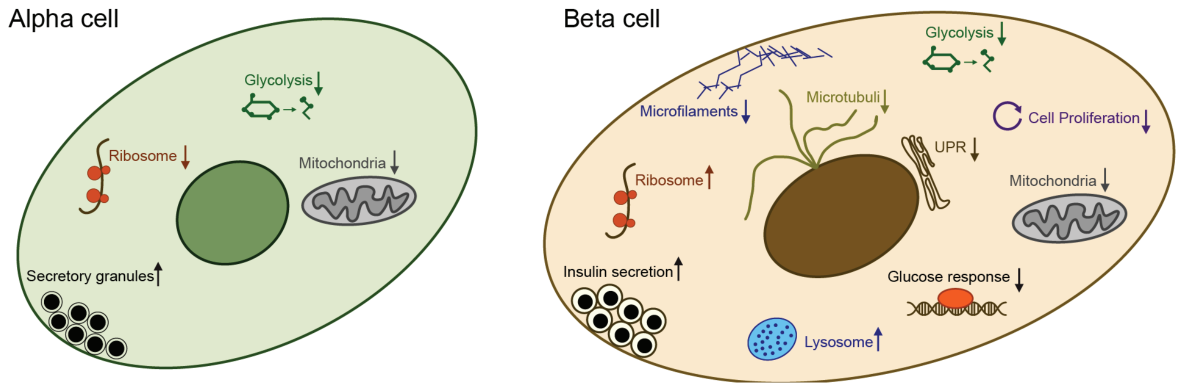

Figure S11. Biological processes affected in T2D alpha- and beta cells.

Hyper-coordinated processes marked with ↑ and de-coordinated processes marked with ↓.
