# Supplementary material for: Single-cell mRNA-regulation analysis reveals cell type-specific mechanisms of type 2 diabetes
Source: Nat Commun. 2025 Oct 27;16:9475. doi: 10.1038/s41467-025-65060-z (PMC12559761; doi:10.1038/s41467-025-65060-z)
Supplement: Supplementary file 2 — Description of Additional Supplementary Files [file 41467_2025_65060_MOESM2_ESM.pdf]

Description of Additional Supplementary Files

Supplementary Data 1: Donor characteristics and cell numbers for each pancreatic cell type in dataset 1 and dataset 2.

Supplementary Data 2: Beta cell dGCNA modules, GO-terms and differentially expressed genes.

Supplementary Data 3: Enrichment of GWAS genes, patch seq genes, and whole genome CRISPR screen genes in dGCNA genes for beta cells and alpha cells.

Supplementary Data 4: mRNA expression of target genes in four large human islet bulkRNAseq datasets.

Supplementary Data 5: Differentially expressed genes and GO-term analysis for *Cebpg* KD INS-1 832/13 cells.

Supplementary Data 6: Alpha cell dGCNA modules, GO-terms and differentially expressed genes.
